# ISOL@: an Italian SOLAnaceae genomics resource

**DOI:** 10.1186/1471-2105-9-S2-S7

**Published:** 2008-03-26

**Authors:** Maria Luisa Chiusano, Nunzio D'Agostino, Alessandra Traini, Concetta Licciardello, Enrico Raimondo, Mario Aversano, Luigi Frusciante, Luigi Monti

**Affiliations:** 1 Department of Soil, Plant, Environmental and Animal Production Sciences, University Federico II of Naples, Portici (NA), Italy; 2 C.R.A. - A.C.M. Centro di Ricerca per l'Agrumicoltura e le Colture Mediterranee, Acireale (CT), Italy

## Abstract

**Background:**

Present-day *‘-omics’* technologies produce overwhelming amounts of data which include genome sequences, information on gene expression (transcripts and proteins) and on cell metabolic status. These data represent multiple aspects of a biological system and need to be investigated as a whole to shed light on the mechanisms which underpin the system functionality.

The gathering and convergence of data generated by high-throughput technologies, the effective integration of different data-sources and the analysis of the information content based on comparative approaches are key methods for meaningful biological interpretations.

In the frame of the International Solanaceae Genome Project, we propose here ISOLA, an Italian SOLAnaceae genomics resource.

**Results:**

ISOLA (available at ) represents a trial platform and it is conceived as a multi-level computational environment.

ISOLA currently consists of two main levels: the genome and the expression level. The cornerstone of the genome level is represented by the *Solanum lycopersicum* genome draft sequences generated by the International Tomato Genome Sequencing Consortium. Instead, the basic element of the expression level is the transcriptome information from different Solanaceae species, mainly in the form of species-specific comprehensive collections of Expressed Sequence Tags (ESTs).

The cross-talk between the genome and the expression levels is based on data source sharing and on tools that enhance data quality, that extract information content from the levels' under parts and produce value-added biological knowledge.

**Conclusions:**

ISOLA is the result of a bioinformatics effort that addresses the challenges of the post-genomics era. It is designed to exploit *‘-omics’* data based on effective integration to acquire biological knowledge and to approach a systems biology view. Beyond providing experimental biologists with a preliminary annotation of the tomato genome, this effort aims to produce a trial computational environment where different aspects and details are maintained as they are relevant for the analysis of the organization, the functionality and the evolution of the Solanaceae family.

## Background

The Solanaceae family comprises about 95 genera and at least 2,400 species. Many of these species have considerable economic importance as food (tomato, potato, eggplant, garden pepper), drug (tobacco) and ornamental plants (petunia). The Solanaceae species show a wide morphological variability and occupy various ecological niches though they share high genome conservation. The need of increasing the knowledge on the genetic mechanisms which determine Solanaceae diversification and adaptation has brought the scientific efforts to be gathered into the International Solanaceae (SOL) Genome Project [1].

The cultivated tomato, *Solanum lycopersicum*, is the plant chosen by the SOL initiative for a BAC-by-BAC sequencing of the genome euchromatin portion, which is considered the gene rich region and represents the 25% of the entire genome [2].

The long term goal is to exploit the information generated by the Tomato Genome Sequencing Consortium [3] for the analysis of the genome organization, the functionality and the evolution of the entire Solanaceae family.

The major bioinformatics effort within the international project currently aims to provide a high quality and homogeneous annotation of the tomato genome. To this end, the international Tomato Annotation Group (iTAG) organized an annotation pipeline which is parcelled out among different members of the consortium and which will result in the release of the annotated tomato genome.

As members of the iTAG, we are committed to collect and manage Expressed Sequence Tag (EST) data from different Solanaceae species and to generate alignments of native and non-native ESTs to the tomato genome.

Furthermore, large amounts of data from different *‘-omics’* approaches are being generated to address key questions risen by the SOL vision. Raw data are hardly useful as they stand and need to be converted into biologically meaningful information. Therefore, bioinformatics approaches become pre-eminent, though their results may be far from being exhaustive and complete. The success of bioinformatics is directly dependent on the efficiency of integration, which in turn is determined by the diversity of data sources, the quality of their annotation and the level of details of the information produced. This remarks the need of designing computational platforms to support research in the post-genome era. The Solanaceae family represents a suitable context to face these challenges and to optimize and tune up the existing bioinformatics methods. To this end, we designed a trial platform, ISOLA (Italian SOLAnaceae genomics resource), for exploiting Solanaceae *‘-omics’* data to address different and complex biological questions crossing the *‘-omics’* barrier for exploring the data as a whole.

In addition, such an effort is going with the iTAG tomato genome annotation, and will maintain details and features that will be reconciled into, or will be discarded by, the official annotation, but that may still highlight useful information. Meanwhile the tomato genome initiative is under way, this platform provides a preliminary annotation of the tomato genome and makes available to the plant community tools for its structural, functional and comparative analyses.

## Results

Here we present ISOLA, an Italian SOLAnaceae genomics resource available at [4]. ISOLA is designed as a multi-level computational environment and meets the need to collect, integrate and explore high-throughput and heterogeneous biological data with the intent that the quality of the data gathered could be enhanced.

**Figure 1:**
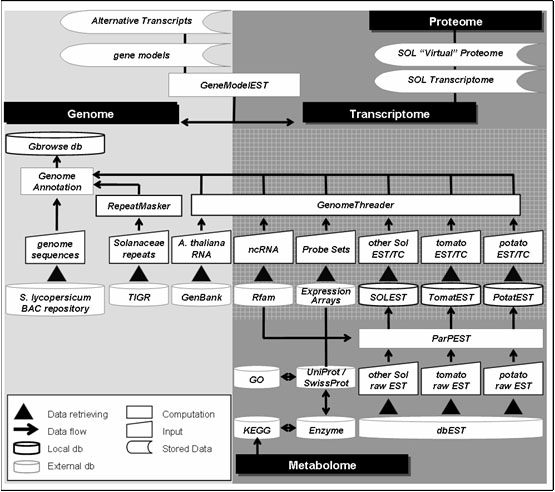
**Representation of the multilevel structure in ISOLA**. Data sources, tools and methods of the platform are indicated. The genome and the expression levels' under parts are included in the light and dark grey areas, respectively. Shared data are located in the gridded area. Subsidiary tools lay on the interface of the two levels. Value-added data gathered from the platform are listed and enclosed in the level to which they contribute more. Entry points for proteome and metabolome approaches are indicated.

ISOLA is currently organized into two main levels: the genome and expression levels (Figure 1). The cornerstone of the genome level is represented by the tomato genome draft sequences. The basic elements of the expression level are the Solanaceae EST collections and the oligonucleotide probe-sets of the tomato expression micro-arrays [5,6].

*‘Basic’* tools are designed and included into the multi-level environment for enhancing data quality and increasing data information content. *‘Subsidiary’* tools lay over the existing multi-level environment exploiting the synergy between the levels.

**Figure 2:**
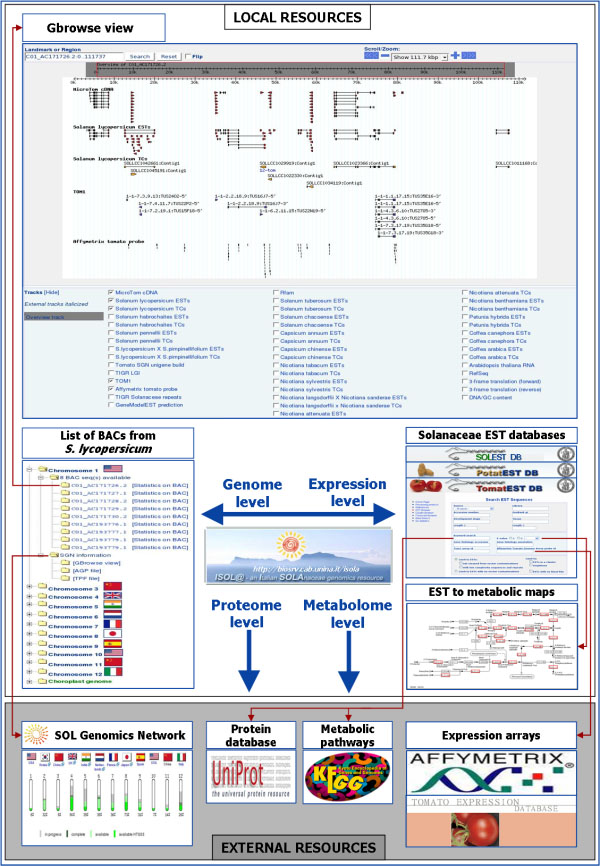
**Snapshot of the Web-based application for navigating ISOLA**. ISOLA is accessible through two different gateways. The Genome Browser gateway let the user explore the list of the tomato BAC sequences, grouped by chromosome number, and visualize the tracks that are displayed along each BAC sequence. Each track is cross-linked to other local or external resources. Cross-references to the tomato genome annotation pages at the SOL Genomics Network are part of the ‘genome level’ too. The Solanaceae EST database gateway let the user investigate Solanaceae transcriptomes as revealed by EST sampling. Each EST database can be queried with keywords to identify functional annotations associated to a single EST or a TC. Cross-links to the UniProt external resource are established. In case an expressed sequence affects an enzyme function, a cross-link to the corresponding KEGG metabolic pathway(s) is provided. These links permit data from proteomics and metabolomics approaches to be integrated in the existing multi-level environment. In addition, the association of the tomato ESTs to the oligo-nucleotide probes from the Affymetrix or from the TED database expands information concerning the ‘expression level’ and provides the opportunity to integrate into the platform data from expression profiling arrays.

Each level can be independently accessed through specific Web applications which allow user-driven data investigation and permit overall as well as detailed views of specific information (Figure 2). A non-stop cross-talk between the genome and the expression levels is based on data source sharing and on tools which accomplish data integration and convergence.

Herein, we describe the organization and the maintenance of the platform which has been designed in order to be extended, through pre-defined entry points, to the proteome and metabolome levels.

### Genome level

#### BAC sequences retrieval

An automated pipeline has been implemented in order to ensure a daily retrieval of new *S. lycopersicum* BAC sequences from the GenBank repository, which are used to feed the genome annotation process. The current collection (May 2007) comprises 129 BAC sequences.

#### BAC annotation

The BAC annotation process aims to identify coding regions and other genetic elements along the *S. lycopersicum* genome sequences.

The protein coding *‘gene finding’* process is exclusively based on the EST spliced-alignments to the genome sequences. To accomplish this task, ESTs from different plant sources (Solanaceae and Rubiaceae species), and the corresponding tentative consensus sequences (TCs), which have been generated by assembling ESTs in a cluster [7], are used. The available data are all described in table 1 where the two rightmost columns (*ESTs/TCs mapped*) report the number of ESTs and TCs per species, aligned to the 129 BAC sequences. ESTs of non-native origin (i.e. EST data compiled in the local PotatEST and SOLEST databases) are included in the analysis so to improve detection of coding regions which lack source-native EST evidence and support comparative approaches.

**Table 1:** Statistics on the EST collections.

family	genus	sub-genus	species	total EST	nr ESTs	gene indices	TC	sEST	total transcripts	ESTs mapped	TCs mapped
Solanaceae	Solanum	Lycopersicum	SOLLC	250552	190763	44759	17629	28005	45634	14286	975
Solanaceae	Solanum	Lycopersicum	SOLPN	8346	6888	3863	730	3140	3870	488	37
Solanaceae	Solanum	Lycopersicum	SOLHA	8000	7868	4101	907	3203	4110	306	30
Solanaceae	Solanum	Lycopersicum	SOLLP	1008	979	744	94	650	744	3	0
Solanaceae	Solanum		SOLTU	226805	206696	62752	19732	44138	63870	5785	584
Solanaceae	Solanum		SOLCH	7752	7750	7192	306	6886	7192	139	10
Solanaceae	Nicotiana		TOBAC	74940	67745	37845	7529	30578	38107	403	74
Solanaceae	Nicotiana		NICBE	27010	24784	9420	3206	6315	9521	95	21
Solanaceae	Nicotiana		NICLS	12448	11749	6785	958	5840	6798	40	6
Solanaceae	Nicotiana		NICSY	8580	8425	7534	512	7023	7535	68	4
Solanaceae	Nicotiana		NICAT	329	324	312	11	301	312	3	0
Solanaceae	Capsicum		CAPAN	31089	28664	15703	3474	12262	15736	373	67
Solanaceae	Capsicum		CAPCH	372	372	343	11	332	343	4	0
Solanaceae	Petunia		PETHY	10670	10336	7004	1166	5842	7008	31	4
Rubiaceae	Coffea		COFCA	46907	38308	16121	4494	11713	16207	20	10
Rubiaceae	Coffea		COFAR	1071	1059	1007	42	965	1007	3	1

**species:** SOLLC: *Solanum lycopersicum*; SOLPN: *Solanum pennellii*; SOLHA: *Solanum habrochaites*; SOLLP: *S. lycopersicum X S. pimpinellifolium*; SOLTU: *Solanum tuberosum*; SOLCH: *Solanum chacoense*; TOBAC: *Nicotiana tabacum*; NICBE: *Nicotiana benthamiana*; NICLS: *Nicotiana langsdorffii x Nicotiana sanderae*; NICSY: *Nicotiana sylvestris*; NICAT: *Nicotiana attenuata*; PETHY: *Petunia x hybrida*; CAPAN: *Capsicum annuum*; CAPCH: *Capsicum chinense*; COFCA: *Coffea canephora*; COFAR: *Coffea arabica*.

**nr EST:** non-redundant ESTs. It is the number of EST sequences obtained after the removal of over-represented ESTs from each collection. **Gene indices** are created by grouping overlapping EST sequences into clusters. Each cluster corresponds to a unique gene. **TCs:** tentative consensus; TCs are generated from multiple sequence alignments of ESTs (assembling process). **sESTs**: singleton ESTs. The **total transcripts** number is obtained adding the TCs to the sESTs. The number of **ESTs** and **TCs** which are** mapped** onto the *S. lycopersicum* genome is included too.

Non-coding RNAs (ncRNAs) from the Rfam collection [8] are aligned to genome sequences too. We identified 105 RNA matches which correspond to 48 different gene *loci*. They represent 10 distinct RNA types whose occurrence and distribution are reported in table 2.

**Table 2:** Occurrence and distribution of non-coding RNA families in the tomato genome draft sequences.

CHR number	BAC accession number	Matching region	Rfam accession number	RNA family	number of matches
1	AC171728.2	17763::18065	RF00017	SRP_euk_srch	1
		135747::135317	RF00005	tRNA	1
1	AC193777.1	99831::99901	RF00005	tRNA	1
4	AC193778.1	148954::149035	RF00005	tRNA	1
4	CT990489.3	73520:73639	RF00086	U27	1
		73740:73863	RF00016	U14	1
4	CU062498.5	60943::B1015	RF00005	tRNA	2
		153111::1S3181	RF00005	tRNA	1
4	CU074307.7	632:702	RF00005	tRNA	1
		153112: 153182	RF00005	tRNA	1
4	CU074307.9	632:702	RF00005	tRNA	1
4	CU074337.8	88091::88161	RF00005	tRNA	1
4	CU104691.12	51151:51223	RF00005	tRNA	2
4	CU179634.6	129509::129931	RF00177	SSU_rRNA_5	44
4	CU222538.4	305::3S6	RF00005	tRNA	1
4	CU222540.3	26296::26383	RF00028	Intron_qpl	1
4	CU2BD045.2	29885::29958	RF00005	tRNA	7
4	CU302231.4	25551::25623	RF00005	tRNA	1
4	CU313315.3	39165::39244	RF00028	Intron_gpl	1
4	CU326362.1	32386::32465	RF00028	Intron_gpl	1
		85446::85520	RF00005	tRNA	2
5	AC194694.1	82814:82839	RF00451	mir-395	1
		82977:83070	RF00451	mir-395	2
		94123::94339	RF00451	mir-395	1
7	AC 187539 1	9990::10061	RF00005	tRNA	1
7	AC187540 1	88984::89055	RF00005	tRNA	1
8	AP009261.1	23923::23995	RF00005	tRNA	1
8	AP009264.1	119184:119256	RF00029	Intron_gpll	2
		123055::123128	RF00029	Intron_gpll	1
		123711-123782	RF00029	Intron_gpll	1
8	AP009267.1	105178::105257	RF00028	Intron_gpl	1
8	AP009274.1	90899::90976	RF00005	tRNA	1
8	AP009279.1	27381::27454	RF00005	tRNA	1
8	AP008280.1	44680::44752	RF00005	tRNA	1
8	AP009281 1	43139:43211	RF00005	tRNA	2
8	AP009284.1	1962:2157	RF00004	U2	1
		121585::121656	RF00005	tRNA	1
8	AP0D9287.1	17284::17354	RF00005	tRNA	1
8	AP009327.1	44492::44572	RF00005	tRNA	1
8	AP009330 1	141496::141567	RF00029	Intron_gpll	1
		142150::142223	RF00029	Intron_gpll	1
		146022:146094	RF00029	Intron_gpll	2
8	AP009357.1	44492::44572	RF00005	tRNA	1
8	AP009360.1	141496::141567	RF00029	Intron_gpll	1
		142150::142223	RF00029	Intron_qpll	1
		146022::146094	RF00029	Intron_qpll	2
10	AC171731.1	9631::9728	RF0G003	U1	1
10	AC193781.1	49869::49939	RF00005	tRNA	1

In a row are reported: the chromosome number; the BAC GenBank accession number; the start/end positions for the BAC regions covered by the RNAs; the Rfam accession number; a brief description of the RNA family; the total number of Rfam sequences per BAC region.

The TIGR Solanaceae Repeats database [9] is the resource selected for the identification of repetitive sequences in the *S. lycopersicum* genome.

The repeats identified on the 129 BAC sequences are listed in Additional file 1 according to the TIGR Plant Repeat Database classification schema. We identified 264 matches corresponding to 71 different genome *loci*. All the genomic regions identified, unless the one detected on the BAC AC171733 (26388::27727) and labelled as unclassified, corresponds to the transposable element (TEs) superclass. Among these 66 are retrotransposons, while the remaining 4 are members of the transposon class. Considering the retrotransposon class, 6 of them are unclassified, 20 are Ty1-copia and 40 are Ty3-gypsy. As usual in plants genomes [10], the transposable elements are ubiquitous and heterogeneous also in tomato. The reason why no other types of repeats have been aligned to the BACs is that the tomato genome sequencing is preliminarily focused on the euchromatic regions [3], which are considered gene richer.

#### The tomato Genome Browser Database

The BAC sequences collected in ISOLA are annotated and released to the scientific community through the Gbrowse [11] Web application at [4]. Tracks showing annotations and other features are displayed and cross-linked to other local or external databases which can be explored through Web interfaces (Figure 2).

#### Aligning *Arabidopsis thaliana* RNA sequences

The availability of the full genome sequence of *Arabidopsis thaliana* is a cornerstone for plant biology [12,13]. We aligned all the RNA sequences from the model plant Arabidopsis to the tomato genome in order to identify genes that are conserved between the two species. However, only 326 out of 31,249 RNA sequences were mapped onto the *S. lycopersicum* BACs.

The majority of the RNA sequences (324) do not overlap any genome region covered by Solanaceae ESTs. The RNA sequences are annotated as tRNAs and locate 24 distinct gene *loci*. The sole AT3G08520 sequence, annotated as “a structural constituent of ribosome”, overlaps *S. lycopersicum* ESTs in correspondence of two different BACs assigned to the chromosome 7. This indicates that, given the large phylogenetic distance between tomato and Arabidopsis, mRNA sequences are hardly identified when the RNA to genome alignments are filtered out with 90% identity and 80% coverage (See Methods).

#### Aligning Affymetrix Tomato Genome Array probe-sets

The Tomato Genome Array is designed specifically to monitor gene expression in tomato and other Solanaceae species [14]. The comprehensive array consists of over 10,000 probe sets to interrogate over 9,200 *S. lycopersicum* transcripts [5].

To date, 4,445 out of 112,528 probes are mapped to the tomato genome. Because some probes are aligned to BAC sequences more than one time, the number of the matches identified (5,827) is higher than the number of the distinct probes aligned.

In particular, 680 oligonucleotide probes do not overlap any *S. lycopersicum* EST. On the other hand 197 probes are in genomic regions where non-native ESTs have been aligned: *S. tuberosum* (164 probes), *S. habrochaites* (12 probes), *S. chacoense* (12 probes) and both *S. habrochaites* and *S. tuberosum* (9 probes). 483 oligonucleotide probes do not overlap any EST.

### Expression level

### EST data processing

We collected all the EST data from Solanaceae species available in dbEST [15] (Table 1). In particular, data are from six species that are members of the genus Solanum of which four from species of subgenus Lycopersicon; five species of the genus Nicotiana; two species of the genus Capsicum and one species belonging to the genus Petunia. We considered also ESTs from two species of the family Rubiaceae genus Coffea, because coffee and tomato share common gene repertoires, as revealed in [16].

A specific *basic* tool has been designed to remove over-represented EST sequences from each of the 16 collections in order to clip the original datasets and produce non-redundant EST collections (Table 1 column 6). These EST collections are independently processed by the ParPEST pipeline [7] in order to i) group ESTs that tag the same gene and generate one *tentative consensus sequences* (TCs) per putative transcript and ii) determine a preliminary functional annotation of both ESTs and TCs.

#### EST sequence databases

**Figure 3:**
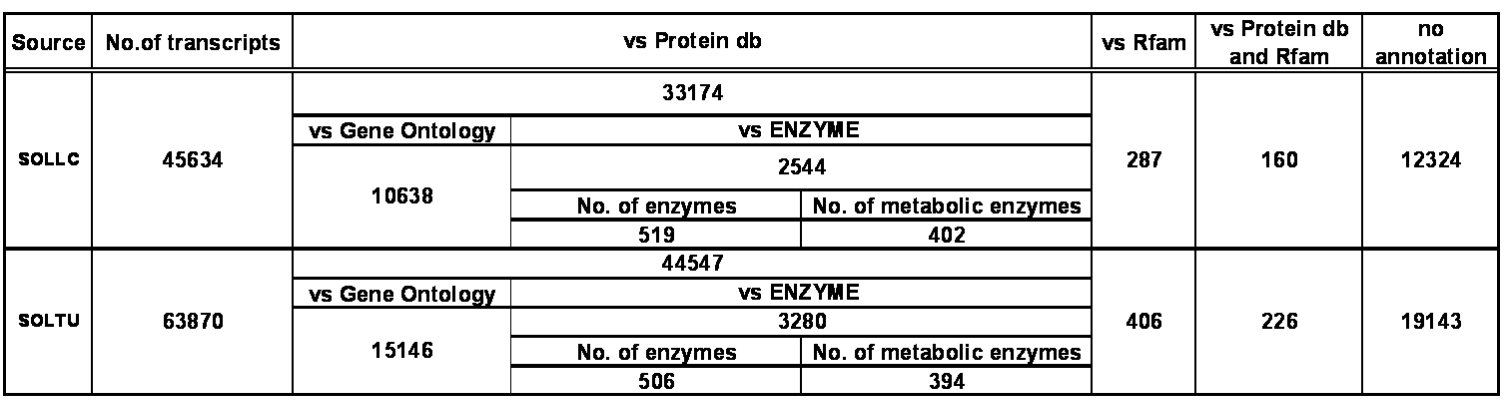
The figure enumerates statistics on the functional annotation versus the UniProt, the Rfam, the Enzyme and the Gene Ontology databases. The number of enzymes involved into known metabolic pathways is reported too.

The EST database architecture is relational. The database stores raw EST sequences and library details; clustering information as well as all the features which describes EST-alignments within clusters. The EST set in a cluster can be assembled into multiple TCs [17] so that their number usually results larger than the number of clusters (Table 1). The total putative transcripts are created by combining the TCs and the singleton EST sequences (sESTs). The putative transcripts are annotated according to similarity versus protein and RNA family databases. A standard classification is provided using the Gene Ontology vocabulary [18] and the Enzyme Commission numbers [19]. Specific Web applications allow transcripts to be dynamically organized into enzyme classes and to be on-the-fly mapped onto the KEGG metabolic pathways [20]. Statistics of different sequence categories per species are reported in table 1. In figure 3 we report information on the functional annotation concerning the most representative transcript collections, i.e. *S. lycopersicum* and *S. tuberosum*.

#### Comparing Tomato Genome Array probe-sets to EST sequences

ESTs from *S. lycopersicum* species are compared to i) the Affymetrix Tomato Genome Array probe-sets [5] and ii) to the EST dataset and the cDNA clones which have been used to build the TOM1 array [6].

Of the 112,528 probes from the Affymetrix Tomato Genome Array, 101,743 have at least one match with an EST sequence. However, all the matches accounts for 735,124 hits.

Considering the 17,015 TOM1 cDNA clones, 9,696 are the number of those sequences that align at least to one EST sequence. The total number of hits is 1,057,491.

### Value-added data-gathering

The higher quality collections obtained by the local data processing are organized into dedicated repositories and are provided to the community as value-added data both through Web applications and FTP services. ISOLA provides: i) the collections of non redundant Solanaceae ESTs and the corresponding computationally defined transcripts, used to sample the species-specific transcriptome space; ii) the definition of the Solanaceae proteomes, including functional annotations and the open reading frame detection, enriching the still poor collection of Solanaceae proteins available from general databases; iii) gene models from tomato, necessary to increase the meagre gene information available to build a training set for gene predictors [20-22]. In order to obtain a reliable and number-consistent collection of gene models, we implemented the GeneModelEST software [24], a *subsidiary* tool in ISOLA.

GeneModelEST requires the genome coordinates of the spliced-alignments of ESTs and TCs which have been independently aligned along the tomato genome draft sequences. Hence, GeneModelEST detects non-overlapping TC sequences which are consistently supported by EST alignments and therefore are evidence of expressed genome regions. These ‘*expressed*’ *loci* are considered to select highly confident gene models. Furthermore, the software exploits the TC functional annotations stored in the EST databases, to check if the ‘*expressed*’ *loci* represent full-length products. Overlapping TCs are explored as they could represent alternative transcripts but are neglected from the selection of gene models because they provide ambiguous information.

In the current update of ISOLA, 339 *S. lycopersicum* TCs have been selected because consistently supported by EST evidence [24] and are displayed as additional tracks in the Gbrowse. Among these TCs, 50 cover at least the 95% of the length of the most similar protein sequence; 96 at least the 50%; 145 cover less than the 50% of the matching protein and the remaining 48 present no significant similarity with any known protein. If the TCs from other tomato species are considered, further 59 *loci* are located. The number accordingly increases to 262 *loci* if the potato TC sequences are also evaluated.

Only the TCs covering the 95% of the length of the matching protein are selected as reliable gene models for the training of gene predictors. They account for a total of 111 gene models.

## Discussion

The success of bioinformatics approaches is directly dependent on the efficiency of data integration and on the value added information which it produces. This is, in turn, determined by the diversity of data sources and by the quality of the annotation they are endowed with.

In the light of these significant issues and in order to approach a systems biology view, we designed a trial computational environment in the framework of the International Solanaceae Genome Project.

ISOLA represents an Italian resource for the genomics of the SOLAnaceae family. It is conceived to collect the overwhelming amount of data that the *‘-omics’* technologies are producing in a quest to investigate multiple aspects of a biological system and tune up bioinformatics methods for novel challenges.

ISOLA consists of data and tools, and aims to meet the need to create a core collection of information from worldwide efforts in order to transform experimental data into biological knowledge. This implies the major effort of enhancing data quality efficiently and of exploiting data integration.

ISOLA is conceived as a ‘data warehouse’ which summarizes information collected periodically from other data systems as well as value-added information generated by the local data processing. The ‘data warehouse’ connects to the external databases from which data are selectively downloaded (Figure 1).

The biological semantic is represented in ISOLA through a multi-level environment which currently consists of two main levels: the genome and the expression levels.

The expression level is mainly represented by EST data collected from libraries of 16 different plant species. These collections are processed by the *basic* tool ParPEST and provide an estimation of the species-specific transcriptome space. The protein-based EST annotation together with the open reading frame detection represents an entry point to plague into the platform data collected from proteomics efforts.

The EST-based collections are organized so to offer the possibility to investigate on different libraries from different tissues and at different developmental stages. The data/tools design permits the study of species-specific expression patterns and their time course, in normal or pathological conditions and/or under specific biotic or abiotic stimula, exploiting the large amount of cDNA libraries today available for tomato, potato and other Solanaceae species. In addition, the link to the KEGG metabolic pathways let metabolome data be integrated in ISOLA so to further support the definition of expression patterns.

Furthermore the genome level is enriched with the annotation of all the gene types (mRNA and non protein coding RNAs), the repeats (simple and complex ones) and other relevant features. In particular, the integration with the wealth collection of pre-annotated data derived from ESTs represents a valuable resource for an efficient analysis and functional annotation of the daily uploaded BAC sequences. In addition, it permits genome-based comparative analyses of the Solanaceae transcriptomes.

To provide a well-founded genome annotation, we are considering only evidence from experimental approaches or from reference databases, neglecting results from gene predictions. Indeed, gene predictors still need to be trained on a consistent set of tomato gene models. The definition of a good quality and representative data set of gene models is one of the tasks of the international Tomato Annotation Group and a preliminary requirement for the training of *ab initio* gene predictors. Gene models represent a value-added information that can be elicited from the effective integration of the main levels of the platform. As an example, GeneModelEST, a *subsidiary* tool in the platform, collects putative alternative transcripts from the ESTs/TCs aligned to the genome and automatically selects high reliable gene models. The availability in ISOLA of expressed sequence collections from other Solanaceae species also contributes to enrich the number of possible coding regions in the tomato genome and to support comparative genomic studies.

The mapping of oligonucleotide probes to the tomato genome as well as to the transcriptome is an entry point to plague into the platform data from expression profiling arrays. This corroborates gene expression studies and supports as the cross-validation of expression patterns derived from microarray analysis, as the detection of network of co-expressed genes derived from EST-based investigations.

The probes-to-genome mapping provides also support for their evaluation. Indeed results demonstrate the non-specificity of some probes and also highlight the presence of probes which are not confirmed by experimentally defined transcripts.

ISOLA can be accessed through two different gateways: the Genome Browser gateway which permits investigations on the BAC sequences and the EST databases gateway for exploring the EST/transcript resources by friendly and flexible interfaces. Both gateways provide access points to the respective levels which are, in turn, cross-linked to support the Web-based navigation (Figure 2).

One of the major efforts in ISOLA is to maintain details of information from all the analytical processes that are part of the platform. This with the purpose to further support the user-driven investigations and the biological knowledge discovery. As an example, in the expression level, raw EST sequence data are maintained to provide the user with information concerning the quality of the cDNA libraries. In order to permit investigations on putative alternative transcripts, the organization of each cluster (made up of ESTs which tag the same gene) into multiple TCs is available. This provides added value that will further support genome investigations.

## Conclusions

ISOLA is based on the collection of the large amount of data produced in the frame of the Solanaceae Genome Project. It represents an Italian effort to integrate heterogeneous data at the semantic as well as at the data source levels. It aims to enhance the quality of the data gathered and to self feed by the value-added information produced.

The platform is becoming a reference within the Solanaceae Genome Project.

ISOLA is daily accessed from scientists from different countries because it provides a preliminary annotation of the tomato genome, while awaiting for the official annotation by the international Tomato Annotation Group. Furthermore, the platform collects and distributes the Solanaceae transcripts, provides their functional annotation and classification, and allows investigations on genome functionalities on the basis of EST supported expression pattern analysis.

Since ISOLA is designed as a multi-level computational environment, it is thought to be flexible and to easily evolve in consideration of the continuous production of new data and novel methods. In addition, ISOLA meets the need to collect, integrate and explore high-throughput biological data in the context of various experiments from multiple organisms. This surely will support successful analysis based on comparative approaches.

Among different platforms for plant genomics, ISOLA represents a novel effort where EST-based functional information is cross-linked to genome data and vice versa, and details are maintained because they are relevant in revealing still hidden biological aspects. Therefore, we believe that the approach here proposed, aimed to support investigations on the structure, the function and the evolution of the Solanaceae genomes, could represent a suitable test bench for similar challenges.

## Methods

### Data sources

1. BAC sequences were downloaded from the GenBank repository.

2. EST sequences were downloaded from the dbEST division of the NCBI.

3. FASTA sequences of Rfam members (version 8.0) were downloaded at . The collection includes 45,644 sequences.

4. The file TIGR_Solanaceae_Repeats.v2 was downloaded from the ftp server . The file is a collection of 252 repetitive sequences reporting transposable, telomere-related, rDNAs and unclassified elements. No centromere-related repeats are included in the collection.

5. The Tomato Affymetrix probe sequence file was retrieved from . The collection includes 112,528 oligonucleotide probes.

6. The TOM1 cDNA microarray sequences data were kindly provided by Jim Giovannoni on March 2006. The collection includes 17,015 sequences.

7. The files NC_003070.frn, NC_003071.frn, NC_003074.frn, NC_003075.frn and NC_003076.frn were downloaded from the genome session of the NCBI ftp server in order to collect all the RNA from *Arabidopsis thaliana*. A total of 31,249 RNA sequences were retrieved.

### BAC sequences retrieval and annotation pipeline

The automated pipeline, that downloads and annotates the BACs which are released to GenBank from the Tomato Genome Sequencing Consortium, is composed of several Perl scripts. Each script enables a specific task inside the pipeline. Using BioPerl an Entrez query is formulated to the NCBI web services system. The query is daily performed to select all the tomato BAC sequences to be retrieved as they represent novel submissions to GenBank or modified records. The pipeline checks, via BAC identifier (i.e. accession.version), if the sequences are already annotated and deposited into the local Genome Browser Database. The retrieved BACs are used to feed into the annotation pipeline.

The mapping of ESTs/TCs, cDNAs, RNAs and expression array probes is performed by GenomeThreader [25]. The parameter settings fixed the percent identity threshold to 90%, the percent coverage to 80% and, in case of EST/mRNAs the poly-A tail masking is applied.

The identification of simple and complex repeats is performed by RepeatMasker [26] using the SGN tomato UniRepeats [27] as filtering database. Two distinct Perl script converts the results of the GenomeThreader as well as the RepeatMasker analysis into the GFF3 [28] format to permit their uploading into the local Genome Browser Database.

### Transcriptome sampling

We used ParPEST [7] to pre-process, cluster, assemble and annotate ESTs from Solanaceae species.

ESTs and TCs are collected into dedicated repositories whose features are described in [17]. The annotation of the expressed sequences is based on the use of controlled vocabularies such as the Gene Ontologies [18] and the Enzyme Commission numbers [19] and the ‘on the fly’ mapping of the expressed sequences [17] onto known metabolic pathways from KEGG [20]. The annotation pipeline includes the analysis of non coding RNAs from RNAfam [8] and, for each tomato EST, a link to the identification numbers of TOM1, the reference cDNA microarray for Tomato [6], and to the probe set of the Affymetrix Tomato Genome Array [5].

## Availability

ISOLA is available at 

## Competing interests

The authors declare that they have no competing interests.

## Authors' contributions

MLC conceived the project, directed its design and implementation, coordinated the different efforts and wrote the manuscript; NDA was mainly involved in the development, the organization and the maintenance of the EST databases and its basic tools and contributed to write the manuscript; AT was mainly involved in the development and maintenance of the Genome Browser database and its basic tools; CL contributed in the data integration effort; ER contributed in the implementation of some tools; MA supported the organization of the EST database and the technical aspects of the platform; LF and LM contributed to the design and the realization of the project. All authors read and approved the final manuscript.

## Supplementary Material

Additional file 1: File format: excel Title: Occurrence and distribution of repetitive DNA in the 129 tomato BACs. Description: The Table includes the chromosome number the BAC GenBank accession numbers the start/end positions for the BAC regions covered by TIGR Repeats the TIGR Repeat class and sub-class and the total number of repetitive DNA sequences per BAC region. Click here for file
